# Occurrence, characterization, and antibiogram of *Staphylococcus aureus* in meat, meat products, and some seafood from Libyan retail markets

**DOI:** 10.14202/vetworld.2019.925-931

**Published:** 2019-06-29

**Authors:** Hesham T. Naas, Ramadan A. Edarhoby, Aboubaker M. Garbaj, Salah M. Azwai, Said K. Abolghait, Fatim T. Gammoudi, Ashraf A. Moawad, Ilaria Barbieri, Ibrahim M. Eldaghayes

**Affiliations:** 1 Department of Food Hygiene and Control, Faculty of Veterinary Medicine, University of Tripoli, P.O. Box 13662, Tripoli, Libya; 2 Department of Microbiology and Parasitology, Faculty of Veterinary Medicine, University of Tripoli, P.O. Box 13662, Tripoli, Libya; 3 Department of Food Hygiene and Control, Faculty of Veterinary Medicine, Suez Canal University, Ismailia 41522, Egypt; 4 Department of Food Hygiene and Control, Faculty of Veterinary Medicine, Cairo University, 12211 Giza 12211, Egypt; 5 Department of Genetics, The Lombardy and Emilia Romagna Experimental Zootechnic Institute, Via Bianchi 9, Brescia 25124, Italy

**Keywords:** 16S rDNA, antibiogram, meat products, meat, seafood, *Staphylococcus aureus*

## Abstract

**Aim:**

The aim of the current investigation was to screen the presence of *Staphylococci* spp., especially *S. aureus* in meat, meat products of different animal species, and some seafood sold in some retail markets in Libya using cultural and molecular techniques, and to study their antibiotics resistance profiles.

**Materials and Methods:**

A total of 139 samples from red meat, meat products, and seafood were collected from many areas in Libya. Enumeration and isolation of *Staphylococci* spp. and *S. aureus* by normal cultural methods followed by molecular identification using molecular techniques by bacterial DNA extraction and partial sequencing of 16S rDNA.

**Results:**

Out of 139 samples, 112 (80.6%) were contaminated with different species of *Staphylococci* based on cultural characteristics of *Staphylococci* on Baird-Parker medium, for which *S. aureus* was detected in only 32 samples (23%). However, only six out of 18 (33.3%) isolates sent for sequencing were confirmed to be *S. aureus* using the molecular technique. The six identified isolates of *S. aureus* were tested for antimicrobial resistance against 24 most commonly used antibiotics. All isolates were resistant to only two antibiotics (cefotaxime and clindamycin). Among these six isolates, only one confirmed to be *Methicillin-resistant Staphylococcus aureus*.

**Conclusion:**

Results of this study suggest that food of animal origin could be a source of *S. aureus* with antimicrobial resistance characteristics that can be spread through the food chain, and raise the importance of these results for public health.

## Introduction

*Staphylococcus aureus* (*S. aureus*) is a Gram-positive bacterium that can cause many diseases that can be minor skin infections or even lethal such as pneumonia and endocarditis [1]. It is a common human pathogen that can be as a commensal flora, and on another aspect, it might be considered as a major cause of some human illnesses [2,3]. *S. aureus* is an opportunistic pathogen that may lead to severe health problems due to its wide antibiotic resistance [4]. Ingestion of staphylococcal enterotoxins produced in food by *S. aureus* enterotoxigenic strains will result in staphylococcal food poisoning that can be considered as one of the most common foodborne diseases. Meat may be contaminated with *Staphylococci* spp. during slaughter or later during the meat preparation [5]. Contaminated food with pathogenic bacteria is one of the main causes of digestive illnesses in developing countries and could be counted as one of the major causes for morbidity and mortality [6]. The presence of *S. aureus* in food can be considered as an indicator for poor hygiene and improper storage conditions [7]. Food processing with poor hygienic practice is highly associated with infection of *S. aureus* enterotoxin [8]. Outbreaks can be contributed to many factors including improper cooking, inadequate preparation of food and contaminated water or raw materials used for food preparation [9]. High level of *Staphylococci* spp. isolation from personnel and equipment in slaughterhouses is reported and correlated when hygiene practice in the slaughterhouses is substandard [10]. Contamination of meat by antimicrobial-resistant bacteria may be the direct cause of foodborne diseases, and as public health importance, this may result in drug resistance of human pathogenic bacteria [11]. *S. aureus* with multidrug-resistant pattern has been reported in many studies [4,12]. Drug resistance in foodborne pathogens may result from the extensive uses of antibiotics [13].

In Libya, all previous studies which were carried out to isolate and identify foodborne *S. aureus* were conducted on samples collected from one area and performed by conventional microbiological methods, with no reports for their antibiogram [14-16]. It is of paramount importance to conduct more studies to reveal the current situation in Libya, to determine the role of the food chain in the transmission of *S. aureus*, and to determine the incidence of *methicillin-resistant S. aureus* (MRSA), which has not been investigated thoroughly and not well documented.

Therefore, the aim of the current investigation was to screen the presence of *Staphylococci* spp., especially *S. aureus* in meat, meat products of different animal species, and some seafood sold in some retail markets in Libya using cultural and molecular techniques, and to study their antibiotics resistance profiles.

## Materials and Methods

### Ethical approval

No ethical approval was required as no live animals were used in this study. However, samples were collected directly from the markets.

### Samples collection and preparation

A total of 139 samples of meat, meat products, and seafood (Tables-1 and 2) were collected from different city markets from different regions of Libya (Tripoli, Regdalin, Sabha, Benghazi, Janzour, and Tobruk). The samples were marked, identified, and packed in sterile bags and transported at 4°C using ice box to the laboratory. All samples were analyzed for enumeration and isolation techniques of total *Staphylococci* and *S. aureus* according to the method described by the International Organization for Standardization (ISO: 6888-1) [17]. Confirmation of *S. aureus* isolates was done by polymerase chain reaction (PCR) and partial sequencing of 16S rDNA.

**Table-1:** Incidence of *Staphylococci* spp. and *S. aureus* in different meat, meat products, and some seafood samples on Baird-Parker medium.

Type of meat	No. of samples	Mean (CFU/g)	No. of positive samples for *Staphylococci* spp. (%)	No. of positive samples for *S. aureus* (%)
Red meat	44	2×10^4^	34 (77.3)	8 (18)
Chicken meat	10	3.3×10^5^	10 (100)	4 (40)
Seafood	19	1×10^6^	11 (58)	1 (5.3)
Red meat products	39	1×10^5^	31 (79.4)	11 (28)
Chicken meat products	27	1.9×10^5^	26 (96.2)	8 (29.6)
Total	139		112 (80.6)	32 (23)

*S. aureus*=*Staphylococcus aureus*

**Table-2:** Incidence of selected *S. aureus* isolated from meat, meat products, and some seafood samples on Baird-Parker medium.

Type of sample	No. of samples	No. of selected *S. aureus* isolates (%)	Mean (CFU/g)	No. of positive *S. aureus* by partial sequencing of 16S rDNA (%)
Beef	15	3 (20)	5.9×10^3^	None
Camel meat	21	5 (23.8)	4.1×10^3^	1 (20)
Chicken meat	10	4 (40)	7×10^3^	None
Mutton	8	None	None	None
Beef burger	12	3 (25)	6.2×10^5^	1 (33)
Beef sausage	16	4 (33)	1.6×10^4^	None
Ground beef	11	4 (36.4)	1.9×10^3^	None
Chicken burger	12	6 (50)	4.2×10^3^	4 (66.6)
Ground chicken	5	1 (20)	9.2×10^3^	None
Chicken sausage	10	1 (10)	1.8×10^4^	None
Mackerel	4	None	None	None
Sardine	7	None	None	None
Clam	4	1 (25)	1×10^5^	None
Shrimp	4	None	None	None
Total	139	32 (23)		6 (18.7)

*S. aureus*=*Staphylococcus aureus*

### Enumeration and isolation of *Staphylococci* spp. and *S. aureus*

Enumeration and isolation of total *Staphylococci* and *S. aureus* were performed using Baird-Parker (BP) agar medium (CM 275, Oxoid, UK) supplemented with egg yolk tellurite emulsion (SR 54, Oxoid, UK). The plates were inoculated with 0.1 mL of appropriate homogenate dilutions using surface plating technique onto BP dry surface plates. Inoculated plates were incubated at 37°C for 24-48 h. Typical black colonies surrounded by opaque halo on BP agar considered as presumptive *S. aureus*. Only plates that can be counted were chosen that contains 25-250 colonies [18].

### Purification of isolated strains

One typical colony of selected *S. aureus* under sterile condition heavily streaked on BP agar and incubated at 37°C for 24 h. Heavy growth harvested and stored using a sterile plastic loop in Eppendorf tube under illuminated sterile cabinet in nutrient broth containing 30% (v/v) glycerol (BDH Chemicals Ltd. Pool, England) then stored at −80°C for further investigation.

### DNA extraction and amplification of 16S rDNA

DNA of *S. aureus* isolates was extracted using the GF-1 bacterial DNA extraction kit (Cat. # GF-BA-100, Vivantis, Malaysia) as reported in a previous study, using same primers to amplify the 16S rDNA [19,20].

### DNA sequencing

The excision of the targeted 16S rDNA PCR fragment (464 bp) from the gel was done. Extraction of the DNA from the gel was carried out using the GF-1 Ambi Clean kit (Cat. # GF-GC-100, Vivantis, Malaysia), as described previously [21]. The purified 16S rDNA amplicons were sequenced in Istituto Zooprofilattico Sperimentale della Lombardia e dell’Emilia Romagna (IZSLER) Institute in Brescia, Italy, and the obtained sequences were blasted.

### Antibiogram assay

On confirmation of *S. aureus*, isolated strains were cultured and prepared for antibiogram assay according to the method described by Coyle [22].

Antibiotics with different classes and commonly used were selected including: Amoxicillin (10 µg), amoxicillin/clavulanic acid (30 µg), ampicillin (10 µg), bacitracin (10 µg), penicillin G (10 µg), methicillin (5 µg), erythromycin (15 µg), gentamicin (10 µg), kanamycin (30 µg), lincomycin (10 µg), tobramycin (10 µg), vancomycin (10 µg), levofloxacin (5 µg), clindamycin (2 µg), cefotaxime (30 µg), doxycycline (30 µg), ciprofloxacin (5 µg), cloxacillin (5 µg), nitrofurantoin (300 µg), oxytetracycline (30 µg), streptomycin (10 µg), tetracycline (30 µg), chloramphenicol (30 µg), and sulfamethoxazole/trimethoprim (25 µg). All antibiotics used were obtained from Oxoid, Hampshire, England, except gentamicin (10 µg) and methicillin (5 µg) were obtained from Bioanalyse®, Turkey.

The determination of antimicrobial resistance patterns of *S. aureus* isolates was done using the agar disk diffusion method and according to the National Committee for Clinical Laboratory Standards criteria [23].

## Results

### Enumeration and isolation of *Staphylococci* spp. and *S. aureus*

The highest mean count of *Staphylococci* was recorded in seafood samples (1×10^6^ CFU/g); while the lowest mean count was in red meat samples (2×10^4^ CFU/g) (Table-1). *S*. *aureus* was isolated from seafood (5.3%), red meat (18%), red meat products (28%), chicken meat (40%), and chicken meat products (29.6%). The mean counts of *S*. *aureus* in different types of samples are shown in Table-2. Meanwhile, mutton, fish, and shrimp samples revealed no growth. The overall incidence of *Staphylococci* in the current study was 80.6% (112/139), and *S. aureus* was 23% (32/112), while the highest incidence recorded in chicken burger 50% and the lowest was in chicken sausage 10% (Table-2).

### Identification of *S. aureus* by PCR and partial sequencing of 16S rDNA gene

**Figure-1:**
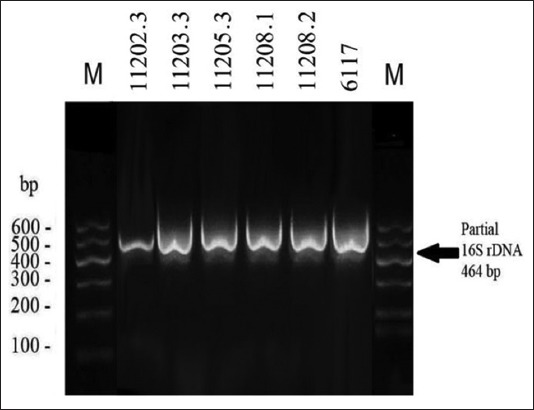
Partial identification of 16S rDNA (464 bp) of isolated *Staphylococcus aureus* strains using the universal oligonucleotides primers. First and last lanes contain the six fragments DNA marker.

Only 18 out of 32 *S. aureus* isolates (Table-3) were sent for partial sequencing of 16S rDNA gene. Only six isolates (33.3%) were confirmed as *S. aureus* (Figure-1). These isolates of *S. aureus* were isolated from camel meat, beef burger, and chicken burger.

**Table-3:** Comparison between conventional and molecular identification of suspected *S. aureus* in different meat, meat products, and some seafood samples.

Type of sample	No. of samples	No. of positive *Staphylococci* on BP	No. of suspected *S. aureus* isolates (sequenced)	No. of positive *S. aureus* isolates by sequencing of 16S rDNA	Incidence of *S. aureus* (%)	

					BP	16S rDNA
Red meat	44	34	8 (4)	1	23.5	25
Chicken meat	10	10	3 (1)	None	30	None
Seafood	19	11	1 (1)	None	9	None
Red meat products	39	31	11 (6)	1	35.5	16.6
Chicken meat products	27	26	9 (6)	4	34.6	66.6
Total	139	112	32 (18)	6	28.6	33.3

*S. aureus*=*Staphylococcus aureus*

### Biochemical results of isolated S. aureus strains

The identified *S. aureus* strains by PCR were examined for their typical biochemical reactions including Gram stain, catalase, oxidase, coagulase, hemolysins and sugar fermentation (mannitol, sucrose, and D-xylose), urease, and novobiocin test.

### Antibiogram results

The six confirmed *S. aureus* isolates were examined against 24 most commonly used antibiotics of different classes for their antimicrobial susceptibility patterns (Table-4) using disk diffusion method and showed different degrees of multi-resistance phenotype. Only five out of 24 (20.8%) antibiotics were effective against all *S. aureus* isolates. In general, only two antibiotics (8.3%) showed complete resistant (100%) by all tested isolates against cefotaxime and clindamycin. Only one tested strain of *S. aureus* isolated from chicken burger showed resistant to methicillin (MRSA).

**Table-4:** Antibiogram results of selected *S. aureus* isolated from meat and meat products samples.

Antibiotics	Isolate code (origin)						R%	S%

	6117 (CM)	11203.3 (CB)	11208.1 (CB)	11205.3 (CB)	11202.3 (BB)	11208.2 (CB)		
Amoxicillin	R	R	R	R	S	R	83.3	16.7
Amoxicillin/clavulanic acid	R	S	S	S	S	S	16.7	83.3
Ampicillin	R	S	S	R	S	S	33.3	66.7
Bacitracin	S	S	S	S	S	S	0	100
Penicillin G	R	S	S	S	R	S	33.3	66.7
Methicillin	I	I	S	I	S	R	16.7	66.7
Erythromycin	S	I	R	R	S	R	50	33.3
Gentamicin	S	S	R	R	S	R	50	50
Kanamycin	S	I	R	R	S	R	50	33.3
Lincomycin	S	S	R	R	S	R	50	50
Tobramycin	S	S	R	R	S	R	50	50
Vancomycin	S	S	S	S	S	S	0	100
Levofloxacin	S	S	S	S	S	S	0	100
Clindamycin	R	R	R	R	R	R	100	0
Cefotaxime	R	R	R	R	R	R	100	0
Doxycycline	S	S	R	R	S	R	50	50
Ciprofloxacin	S	S	I	I	S	I	0	50
Cloxacillin	I	I	R	R	R	R	50	16.7
Nitrofurantoin	S	S	S	S	S	S	0	83.3
Oxytetracycline	I	I	I	I	I	R	16.7	16.7
Streptomycin	R	R	R	R	R	I	66.7	0
Chloramphenicol	S	S	S	S	S	S	0	100
Sulfamethoxzole/Trimethoprim	S	S	S	S	S	S	0	100
Tetracycline	S	S	R	R	R	R	66.7	33.3
R%	29.3	16.7	41.7	54.2	25	54.2		
S%	58.3	62.5	45.8	33.3	70.7	37.5		

CA=Camel meat, CB=Chicken burger, BB=Beef burger, R=Resistant, S=Sensitive. *S. aureus*=*Staphylococcus aureus*

## Discussion

The current investigation was designed to enumerate, isolate, and identify *S. aureus* using conventional culture methods and also by molecular technique by partial sequencing of 16S rDNA and to study antibiotics resistance profile of isolated strains from several samples of meat, meat products, and some seafood samples in Libya. The count of *S. aureus* in meat, chicken meat, seafood, and their products varied depending on different causes such as mishandling, freezing, and food additives.

However, the occurrence of *S. aureus* was only in 32 (23%) samples using a conventional culture method. However, using molecular identification, only six isolates out of 18 sent for sequencing were confirmed to be *S. aureus* (33.3%) with the homology of 99-100%. This is the first study in Libya to confirm the presence of *S. aureus* using the molecular technique.

Red meat samples results were in accordance with Libyan *S. aureus* limit [24]. *S. aureus* isolated from many food products, including meat products, as reported in many studies, to be as a potential risk for public health [25]. In comparison with other reports, coagulase positive *S. aureus* was detected in 16% of the meat samples marketed in Casablanca, Morocco [26]. The results obtained in this study were in agreement with the study of Martins *et al*. [27]. In Egypt, out of 100 staphylococcal isolates from chicken and beef raw meat samples, *S*. *aureus* (15/100) and *Staphylococcus* *epidermidis* (14/100) were the highest incidences [28]. However, in Sudan, in Khartoum State, the frequency of isolation of *Staphylococci* was higher in Omdurman city 23 (39.7 %), whereas in Khartoum city was 17 (29.3%) [29]. Another study from Iran reported that the highest prevalence of *S. aureus* was found in lamb meat (68.8%) in counter with our study, while in beef samples were 57.5% and in camel meat was 46.0% higher than our results; meanwhile, in goat meat was 47.5% [30]. Hanson *et al*. [31] reported that before evisceration, only 6.5% of the carcasses were contaminated with coagulase-positive *S. aureus* compared to 40% carcasses contamination after evisceration.

In relation to the isolation of *S. aureus* from camel meat in Libya, the mean counts were lower than counts of *S. aureus* in camel meat in Esfahan, Iran, but nearly agreed with the estimated count in Jordanian camel meat (4.3×10^2^ and 2.58×10^4^ CFU/g) [30,32], respectively. Meanwhile, the prevalence of *S. aureus* in meat was 24.6% from samples of domestic and imported meat from local retail markets in Riyadh area in Saudi Arabia [33].

As it is well known that cooling and freezing temperatures can help improve safety as well as prolong the shelf life of meat and poultry products by delaying or inhibiting the growth of microorganisms, *Staphylococci* appear to be relatively resistant to the adverse effects of freezing [34].

As collected samples were frozen or chilled raw products, and according to our experience, the high incidence of *S. aureus* in most meat products could be attributed to that local manufacturers use untreated additives and spices, mishandling and/or during the addition of additives without proper hygiene. Furthermore, the microbiological quality of beef burger in fast food restaurants in Tripoli city was investigated, and the results showed that 27.1% of the 59 uncooked samples and 3.2% of the 92 cooked samples were contaminated with *S. aureus* [14]. In addition, out of 511 of street foods in Accra, Ghana, 163 samples contained *S. aureus* (31.9%) [35] nearly similar to our obtained results. Previous studies carried out in Tripoli city, Libya [15-16] detected *S. aureus* in beef burger and beef sausage with a mean count of 5.8×10^5^ and 2×10^5^ CFU/g, respectively, which showed higher mean count than this study. The presence of this pathogen is of primary concern, as it is able to produce heat-stable enterotoxins, and as a subsequent may cause food poisoning in humans [36]. Variation of *S. aureus* means count in meat products might be due to different levels of hygiene during the production and method of preparation.

Seafood and fish can be considered as an excellent substratum for microbial growth due to their high protein levels and high water contents [37]. *Staphylococci* are excluded to be part of the normal fish microflora, and the presence of *Staphylococci* on fish is an indicator for poor personal hygiene for involved people, new contamination, or could be as a disease in fish [38]. The incidence of *Staphylococci* in seafood in our study was 58%, whereas it was 43% in Greece [39].

Only six out of 18 sequenced isolates (33.3%) were confirmed as *S. aureus* using the molecular technique. In another study [40], *S. aureus* was identified as 62.2% positive by PCR compared to 57.7% using the cultural technique. *S. aureus* was isolated from 40 samples with higher percentage when using traditional methods of isolation compared to a lower percentage when using PCR method, with a sensitivity rate ranged from 72 to 82% [41].

In another study, Tassew *et al*. [42] showed that 20 *S. aureus* isolates, resistance to oxacillin (90%), ampicillin (85%), erythromycin (65%), amoxicillin (60%), streptomycin (35%), and vancomycin (20%), and all isolates were sensitive to co-trimoxazole (100%). Hiroi *et al*. [43] reported that *S. aureus* isolated from chicken was resistant to ampicillin (39.2%) and tetracycline (29.1%), whereas *S. aureus* isolated from beef was resistant to ampicillin (59%) and tetracycline (6.8%). Waters *et al*. [12] mentioned that resistance was highly prevalent to tetracycline, ampicillin, penicillin, and erythromycin. In general, the high resistance to each of ampicillin, tetracycline, erythromycin, and penicillin appears clearly in many studies in a ratio ranged from 66% to 85% [44]. All tested *S. aureus* isolates in this study showed 100% resistance to cefotaxime and clindamycin. The difference in the percentage of antibiotic susceptibility may be due to the type of antibiotic used or due to local *Staphylococci* and *S. aureus* strains to each country. In this study, none of the *S. aureus* tested was resistant to vancomycin. In Japan, food samples collected from 2004 to 2006 were analyzed, and the isolation rates of *S. aureus* were 32.8%, in which MRSA was isolated from 3% of meat samples [43]. Boost *et al*. [45] isolated MRSA from 21.9% of pork, 6.8% of chicken, and 4.4% of beef samples.

## Conclusion

Our results showed that isolation of *S. aureus* from Libyan food of animal origin represents important aspects of food safety. The high incidence of *S. aureus* in meat products could be attributed to poor hygienic practice, contaminated additives, and cross-contamination during the preparation of such products. All *S. aureus* isolates confirmed by PCR technique tested for their antibiotic sensitivity profiles showed different degrees of multi-resistance phenotype. This suggests food products of animal origin could play a role in the spreading of *S. aureus* through the food chain with public health concern related to antimicrobial resistance characteristics. Although the frequency of isolation of MRSA is low in this study, our findings demonstrated that food of animal origin contaminated with MRSA may constitute a health hazard to consumers. Therefore, periodic surveillance of drug resistance of these organisms in the food of animal origin in different geographical areas is needed. Finally, the proper application of good manufacturing practice, good hygiene practice, and well-designed hazard analysis of critical control point program in the slaughterhouses and processing units.

## Authors’ Contributions

AMG, SMA, SKA, AAM, FTG, and IME designed and planned this research work. HTN, RAE, AMG, SMA, SKA, AAM, FTG, and IME were involved in the research by collecting samples and doing the laboratory work. IB has carried out the sequences of the PCR products in her laboratory (IZSLER Laboratory in Brescia, Italy). All authors contributed equally in the preparation and revision of the manuscript. All authors read and approved the final manuscript.
